# 

**Published:** 1889-01-19

**Authors:** 


### CONTENTS.

**The Power of the Penny** - 243

**In Spare Moments** - 244

**The Doctor's Pulpit.**—"All Sorts and Conditions" of Women - 245

**Metropolitan Hospitals Ancient and Modern.** — IV. Out and In Patients and the Pay System - 246

**Turning Over New Leaves** - 247

**Voyages in Vacation.**—XV. Russian Dinners, Hotels, Restaurants and Amusements - 249

**Notes and News** - 250

**Everybody's Page** - 252

**Annotations.**—Desperate Diseases, Desperate Remedies—Impossible!—A Sheffield "Scare" - 253

**Babies.**—III. Full Quivers - 254

**Within the Wards.**—Bleeding Revived—A Happy Result, etc. - 255

**False Impressions.** By CAROLINE FOTHERGILL, Author of "An Enthusiast," "A Voice in the Wilderness," etc. - 256

**The Blackheath and Charlton Cottage Hospital** - 257

**Scraps and Gleanings** - 258

**Amusements and Relaxation** - 258

**EXTRA SUPPLEMENT—THE NURSING MIRROR.**

**En Passant.** Short Items—Dissatisfied Probationers—Verses, or Vilification?—District Nurses at Glasgow—Qui Non Proficit, Deficit.—Growls and Grumblings.—Nurse or Mother?—The Order of St John - lxi

**Original Articles.**—A Month in Hospital - lxii

**The Entertainment at St. Bartholomew's** - lxiii

**Are Nurses' Short-lived?—Interviewing a Matron** - lxiii

**The British Nurses' Association** - lxiii

**Everybody's Opinion.**—British Nurses' Association, etc. - lxiv

**Appointments.—Vacancies.—Notes and Queries** - lxiv

